# Veterinary Register—Pennsylvania (*Continued*)

**Published:** 1902-03

**Authors:** 


### VETERINARY REGISTER.

[See Editorial, Journal of Comparative Medicine and Veterinary Archives, July, 1901, page 438.]

[In the following list the names and data printed without comment are those furnished to us by postal card with the signature of the individual.

Those with an asterisk [*] are those to whom we have sent, by United States mail, an envelope with prepaid postage and a request for return if not delivered in ten days; as the envelopes have not been returned, we presume that the letter has been delivered, that the individual in question exists, but that he has neglected to return the enclosed postal card with the information requested.—Ed. Journal.]

### PENNSYLVANIA.

(Continued from page 816, December, 1901.)

| *Name.* | *Street.* | *Town.* | *College, Graduated.* | *County, Registered.* |
|---|---|---|---|---|
| Radley, Aaron W. | Broad & High | Bethlehem | American Vet., 1886 | Northampton |
| Ralston, Lewis | Bridge st | Phœnixville | * | Luzerne |
| Ramson, J. B. | S. Franklin st | Titusville | * | Crawford, 1890 |
| Ranck, Edward M. | 422 N. 41st st | Philadelphia | V. D. U. Pa., 1897 | Philadelphia, 1897 |
| Rayner, G. B. | 141 Fountain av | Manayunk | * | ...... |
| Rayner, Jas. B. | 135 E. Gay st | West Chester | Phila. C. V. S., 1864 | Chester, 1889 |
| Rayner, Thos. B. | Highland av | Chestnut Hill | * | ...... |
| Rayner, Thos. C. | Chelten av | G't'n, Phila. | * | ...... |
| Raynor, John B. | ...... | Branchtown | * | ...... |
| Reading, A. J. | ...... | Hatboro | * | ...... |
| Reagan, W. J. | ...... | Tullytown | * | ...... |
| Rechtenwald, J. J. | 36 Southern av | Mt. Oliver | * | ...... |
| Rechtenwald, N. | 89 Washington | Pittsburg | * | ...... |
| Reed, J. O. | ...... | Danville | * | ...... |
| Reed, J. V. | ...... | Hookstown | * | ...... |
| Reid, Walter | ...... | Shadeland | * | ...... |
| Reig, Vincent | ...... | Carrolltown | * | ...... |
| Reigert, Levi | ...... | Annville Twp. | * | ...... |
| Reinecker, J. W. | ...... | Wellsville | * | ...... |
| Reish, Samuel | ...... | Pleasant Gap | * | ...... |
| Remaley, John | ...... | Nazareth Twp. | * | ...... |
| Rennells, Benj. | ...... | Coudersport | * | ...... |
| Renshimer, V. | ...... | Pipersville | * | ...... |
| Rentschler, H. K. | ...... | Shartlesville | * | ...... |
| Repine, Richard | ...... | Mahoning Twp. | * | ...... |
| Retten, Harvey, M. | ...... | Ephrata Twp. | * | ...... |
| Rhoads, John M. | ...... | Barbara | * | ...... |
| Rhoads, Warren L. | 11 E. Baltimore | Lansdowne | American Vet., 1893 | Delaware, 1893 |
| Ribble, T. D. | ...... | Washington Twp. | * | ...... |
| Rice, Horace | ...... | Union City | * | ...... |
| Rice, Robert G. | ...... | Towanda | * | ...... |
| Richards, H. S. | 6220 Penn av | Pittsburg | Ontario Vet., 1887 | Allegheny, 1890 |
| Richards, John | ...... | Logan Twp. | * | ...... |
| Richards, John M. | ...... | Reading | * | ...... |
| Ridge, Wm. Hodgson | ...... | Trevose | Univ. of Pa., 1888 | Bucks, 1889 |
| Riggle, Jos. J. | ...... | Loretto | * | ...... |
| Riker, N. D. | ...... | W. Nicholson | * | ...... |
| Ripple, Theo. | ...... | Edinburg | * | ...... |
| Riter, John | ...... | Centre Hall | * | ...... |
| Ritter, Chas. | 1722 N. 19th st | Philadelphia | * | ...... |
| Ritter, Henry | ...... | Balliettsville | * | ...... |
| Roberts, Amos B. | ...... | Blandon | * | ...... |
| Roberts, Enoch | ...... | Fairfield Twp. | * | ...... |
| Roberts, Owen B. | ...... | Blandon | * | ...... |

| *Name.* | *Street.* | *Town.* | *College, Graduated.* | *County, Registered.* |
|---|---|---|---|---|
| Robinson, Ephraim | ...... | Clearville | * | ...... |
| Robinson, Hiram | ...... | Robinsonville | * | ...... |
| Robinson, Jas. B. | ...... | Montgomery Twp. | * | ...... |
| Robinson, Wm. | ...... | Broughton | * | ...... |
| Robinson, Reynolds | ...... | Piney Creek | * | Bedford, 1891. |
| Rodgers, Jas. A. | ...... | Weaversville | * | ...... |
| Roehrig, Wm. | 2934 Girard av | Philadelphia | * | ...... |
| Rogers, H. G. | ...... | Washington | * | ...... |
| Rohn, J. P. | 212 N. 15th st | Philadelphia | * | ...... |
| Rohrer, Isaac S. | ...... | Kingers | * | ...... |
| Roll, Albert J. | ...... | Natrona | * | ...... |
| Rook, W. G. | ...... | Scranton | * | ...... |
| Rook, Wm. | 124 Linden st | Scranton | * | ...... |
| Rose, Warren | ...... | Rutland | * | ...... |
| Rosenberry, John G. | ...... | Skippack | * | ...... |
| Ross, Jas. T. | Frankford av and Allen st | Frankford | * | ...... |
| Roth, Philip | ...... | Minesite | * | ...... |
| Rotz, Benj. F. | ...... | Mowersville | * | ...... |
| Rowe, W. A. | 1156 Franklin st | Reading | * | ...... |
| Roxberry, G. W. | "Owens Track" 72d & Island rd | Philadelphia | * | ...... |
| Royer, G. Z. | ...... | Millbach | * | ...... |
| Ruhl, J. S. | ...... | Tylersville | * | ...... |
| Rundle, Wm. L. | ...... | Chapman Quarries | * | ...... |
| Ryder, Daniel | 134 N. Main st | Chambersburg | Ontario Vet., 1890 | Franklin, 1899 |
| Sallade, J. Gill | ...... | New Ringgold | Ontario Vet., 1894 | Schuylkill, 1894 |
| Sallade, James W. | 2d & Orchard | Auburn | Ontario Vet., 1883 | Schuylkill. 1889 |
| Sands, Harry G. | Main & Everett | Benton | Ontario Vet., 1893 | Columbia, 1894 |
| Sasse, E. F. | ...... | Mars | * | ...... |
| Saylor, Jos. | ...... | Fayetteville | * | ...... |
| Schaeffer, C. D. | ...... | Allentown | * | ...... |
| Schaeffer, Jas. D. | ...... | Fleetwood | * | ...... |
| Schafer, Levi | ...... | Greble | * | ...... |
| Schall, H. B. | ...... | Rural Valley | * | ...... |
| Schantz, A. L. | ...... | Allentown | * | ...... |
| Schauffler, Chas. | 915 W. Dauphin | Philadelphia | * | ...... |
| Scheuldt, Ed. | 921 Franklin st | Reading | * | ...... |
| Schlegel, Alfred | ...... | Moore's Twp. | * | ...... |
| Schlegel, Daniel | ...... | Moore's Twp. | * | ...... |
| Schleicher, L. F. | ...... | Riegelsville | * | ...... |
| Schmoyer, W. | ...... | Breinigsville | * | ...... |
| Schneider, F. H. | 3512 N. 11th st | Philadelphia | National Vet., 1896 | Schuylkill |
| Schoch, J. C. | ...... | Middleburg | * | ...... |
| Schireman, A. E. | ...... | Dushore | * | ...... |
| Schreiber, A. F. | 62d & Elmwood | Philadelphia | * | ...... |
| Schroeder, G. P. | ...... | Bower's Station | * | ...... |
| Schultz, Jos. S. | ...... | Zionsville | * | ...... |
| Sebastian, Geo. B. | ...... | Rehrersburg | * | ...... |
| Secrist, Samuel | ...... | Warren Twp. | * | ...... |
| Sedan, Simon, R. | ...... | Delaware Twp. | * | ...... |
| Seeley, Oscar, | Aldine Hotel | Philadelphia | * | ...... |
| Seitter, J. B. | 3528 Kensington | Philadelphia | * | ...... |

